# Notes and News

**Published:** 1896-10-10

**Authors:** 


### NOTES AND NEWS.

(Collected and Communicated.)

Professor Michael Foster delivered the first Huxley lecture at Charing Cross Hospital Medical School on October 5th. The lecture was of the character of an inaugural address. Professor Foster compared the medical curriculum of Huxley's time with that in use at the present day, and pointed out that the sciences of physics, biology, chemistry, and physiology had hardly any place in the former. He illustrated the complexity of the progress of science, and the intricacy of the bearings of even a single scientific observation, by relating the history of four discoveries and the important results which followed in each case. The lecturer concluded by referring to the great influence which had been exerted by Huxley on the study of physiology, and so on that of medicine.

A meeting was held on October 1st at the rooms of the Medical Defence Union to consider the question of hospital abuse, and to decide whether an association should be formed for its suppression. Dr. Ward Cousins took the chair, and among those present were Dr. Horder, Mr. Nelson Hardy, Dr. Sibley, Lieut.-Colonel Montefiore, Dr. Henry, Dr. Hugh Woods, and others. Dr. Horder said that a considerable number of the medical profession agreed with him as to the necessity of some action being taken for the suppression or mitigation of hospital abuse, which, especially in the out-patient department, was greater now than it ever was before. Dublin stood at the head of the list in regard to the proportion of the population who were recipients of hospital relief, Liverpool being the second in this respect, and London the third. He moved a resolution in favour of the formation of a Hospital Reform Association. This was seconded by Dr. T. R. Atkinson, and was carried. Mr. Nelson Hardy was of opinion that the work would be better done by a larger and more powerful body, such as the British Medical Association, and others thought that the matter was one rather for the Charity Organisation Society or for some Central Hospital Board. But the general feeling seemed to be that while some of these bodies had had their opportunity with but small results, they would all be so occupied in other sorts of work that they would not be likely to enter upon this matter with the same effect as an association devoted to the single purpose of preventing hospital abuse.

The foundation-stone of the Hope Hospital, Dumfriesshire, has just been laid by Miss Jane Hope, of New York, sister of the donor, Mr. Thomas Hope, who bequeathed £100,000 to build and endow this hospital for his native town. The object of the bequest appears to be more in keeping with ancient forms of charity than with the hospital proper of the present day, as it is intended not only to benefit the sick, but the aged and infirm in reduced circumstances. It is not quite clear to what extent these latter will be received into the hospital. The accommodation is for fourteen patients, and is estimated at a cost of £17,000.

The winter session at the National Hospital for Paralysis was opened on October 6th by an introductory address by Dr. Gowers, which, however, owing to his unavoidable absence, was read by Dr. Beevor. He drew attention to the great educational advantages offered by the National Hospital for Paralysis, owing to the extraordinary collection of cases of nervous disease always to be found within its walls, and he announced that the hospital now took rank among the officially recognised places of medical education, attendance in its wards during the fifth year of study being now accepted as part of the curriculum by the examining boards. It was impossible to over-estimate the importance of a careful study of diseases of the nervous system, touching as they did upon those of every other part of the human frame. Many diseases, however, the attacks of which showed themselves by symptoms connected with the nervous system, were in origin connected with other systems, and the key to their elucidation lay in a due appreciation of other conditions. The differentiation, for example, between some cases of hæmorrhage in the cerebral substance and cases of thrombosis of a cerebral artery was to be obtained far more by a consideration of the state of the heart and the blood vessels, and of other associated conditions, than from the most careful analysis of the purely nervous derangements produced by the lesion. In regard to many cases also it was necessary to bear in mind that disease, just the same as nutrition, was dependent upon chemical change. The minutest quantity of atropine evenly diffused throughout the whole mass of the blood was selected only by certain tissues, so as to affect the eye while the rest of the body was left untouched. Curare also was picked out by the nerve endings within the muscles, while the rest of the nervous system was unaffected. It must be as the result of some peculiarity in the chemical constitution of the molecules of those tissues that these things happened, and by the products of some resulting decomposition that their function was disturbed. Now, if we looked at the action of certain toxins we found the same selective power, and it might be said that wherever symptoms pointed to an affection of one particular tissue or one particular form of nervous structure throughout the body, there was a strong presumption of the malady being due to the result of the chemical action of some form of toxin. Symmetry of distribution then came to be strong evidence of toxic origin. The complete change in our conception of the "mechanism" of the nervous system which had arisen from modern research, according to which nerve cells were not originators of nerve energy but merely centres of nutrition, was then referred to, and, finally, much useful advice was given as to the best methods of taking notes and recording observations.

